# Dental and craniofacial characteristics in a patient with Dubowitz syndrome: a case report

**DOI:** 10.1186/1752-1947-5-38

**Published:** 2011-01-27

**Authors:** Andrea Ballini, Stefania Cantore, Domenica Tullo, Apollonia Desiate

**Affiliations:** 1 Department of Dental Sciences and Surgery, University of Bari 'Aldo Moro', Bari, Italy

## Abstract

**Introduction:**

Dubowitz syndrome is a very rare, autosomal recessive disease characterized by microcephaly, growth retardation, a high sloping forehead, facial asymmetry, blepharophimosis, sparse hair and eyebrows, low-set ears and mental retardation. Symptoms vary between patients, but other characteristics include a soft high-pitched voice, dental and craniofacial abnormalities, partial webbing of the fingers and toes, palate deformations, genital abnormalities, eczema, hyperactivity, preference for concrete over abstract thinking, language difficulties and an aversion to crowds.

**Case presentation:**

We describe the craniofacial and dental characteristics of a 12-year-old Caucasian Italian boy with both the typical and less common findings of Dubowitz syndrome.

**Conclusion:**

Diagnosis of Dubowitz syndrome is mainly based on the facial phenotype. Possible conditions for differential diagnosis include Bloom syndrome, Smith-Lemli-Opitz syndrome, and fetal alcohol syndrome. As there are few reports of this syndrome in the literature, we hope this case report will enable health professionals to recognize the phenotypic alterations of this syndrome, and allow early referral for the necessary multidisciplinary treatments.

## Introduction

Dubowitz syndrome (DS) was initially confused with Bloom syndrome until it was recognized as a separate condition in 1971 [1]. The two conditions share the common features of pre-natal and post-natal growth failure, microcephaly, high-pitched voice, skin changes (eczematous), cancer predilection and immune deficiency [1]. Children with DS have an unusual facial appearance, mental disability with hyperactivity, and feeding problems during infancy, with overt or submucous cleft palate in 35% of patients [1,2]. Progressive scoliosis [3] and achalasia [4] have also been reported. Neoplasms associated with DS include leukemia, lymphoma, and neuroblastoma [5].

DS is an autosomal recessive condition (OMIM database entry no. 223370) [6]. The specific gene mutation responsible for DS has not yet been identified.

Preventive management of DS should include monitoring of early feeding, evaluation of hypospadia or cryptorchidism (present in 70% of boys with DS), audiology and middle ear examinations, and intervention in early childhood to optimize potential in children with an IQ of 80-90 [5]. Intelligence varies from severe retardation to average levels. Developmental disabilities include delayed speech (60%) and hyperactivity (40%) [1,2,7]. Peripheral blood counts should be obtained in children with infectious illness or fatigue, as aplastic anemias have also been described [1,2].

The objective of this report was to clarify the particular characteristics of a patient with DS, and highlight the importance of early recognition of this condition.

## Case presentation

A 12-year-old Caucasian Italian boy with DS presented to our institution for a dental examination. The diagnosis of DS had previously been made at a hospital where a multidisciplinary group was monitoring our patient.

As stated earlier, the facial phenotype is the primary basis for diagnosis of DS. When we examined our patient, we found a number of clinical anomalies, including a small head, frontal bossing, low-set ears, saddle nose, triangular face, and the characteristic facial features of DS such as palpebral ptosis, hypertelorism and micrognathia. Multiple nevi were also seen. Mild mental retardation was present, and our patient's voice was high-pitched.

We reviewed our patient's medical history. Laboratory studies carried out included routine blood counts and chemistry tests, thyroid function tests, a sweat test and chromosomal analysis, which had all given normal results. Our patient had previously undergone surgery to correct a cleft palate. Congenital intra-atrial and intra-ventricular defects were present, such as a cataract that was also treated surgically. Radiographs of the lumbar column indicated a right convex scoliosis. However, some of the previously reported anomalies in DS were absent.

**Figure 1:**
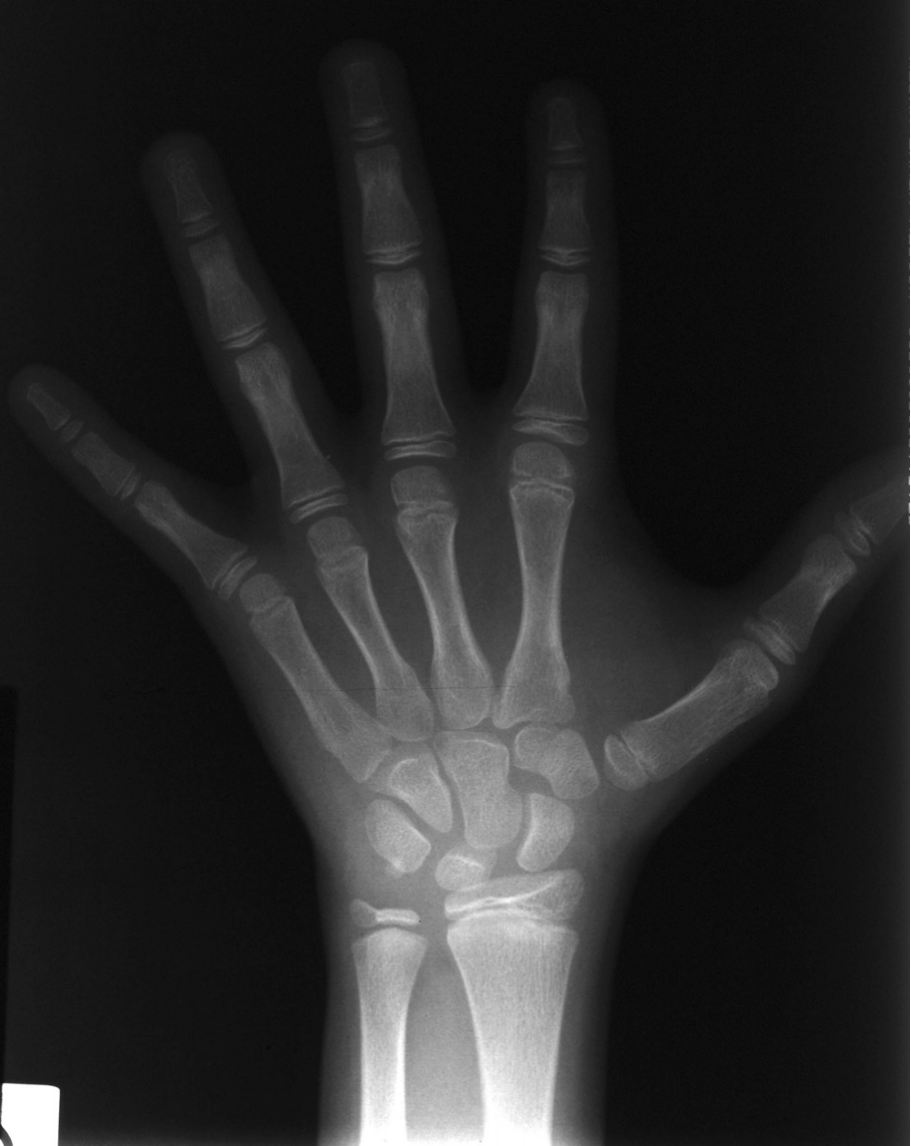
**Hand and wrist X-rays, with bone age corresponding to chronological age**.

**Figure 2:**
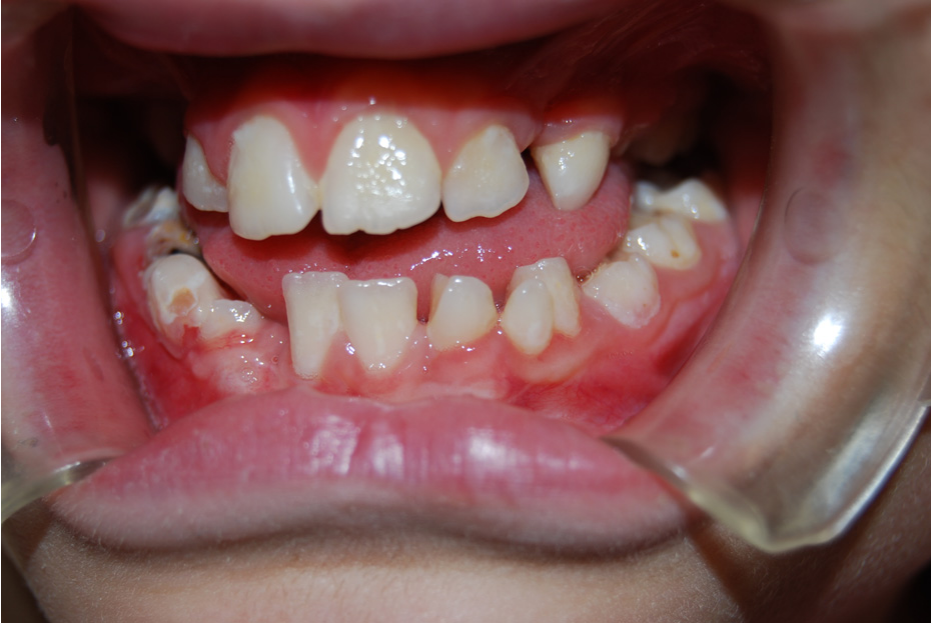
**Absence of lower and the upper right canines, delayed eruption and rotation of the lower incisors incipient cavities**.

**Figure 3:**
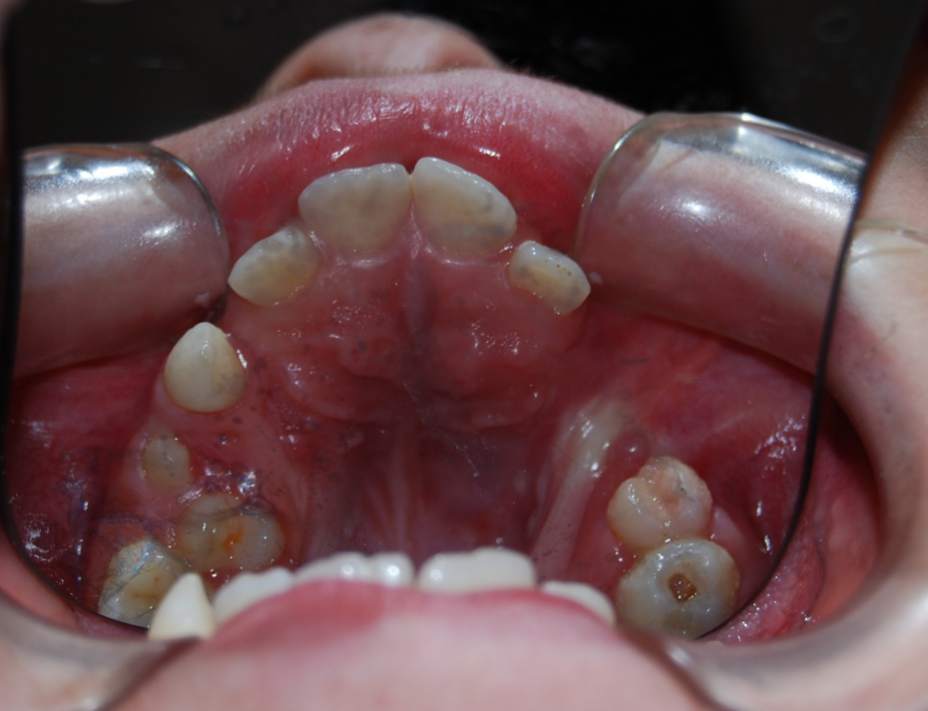
**Narrow palate (previous surgical procedure for cleft palate)**.

During the odontological evaluation we took a full medical history from our patient, and he underwent clinical and imaging examinations. The radiological examination included panoramic, anteroposterior and lateral views of the skull, and hand and wrist radiographs. We also carried out an auxological examination, and found that our patient's bone age was compatible with his chronological age (verified by assessment of the radiographs of the hand and wrist), but his ponderal growth was delayed (Figure 1). The oral clinical examination revealed the absence of both the lower and upper right canines, delayed eruption and rotation of the lower incisors due to deciduous incipient cavities (Figure 2), and a high, narrow palate (Figure 3).

**Figure 4:**
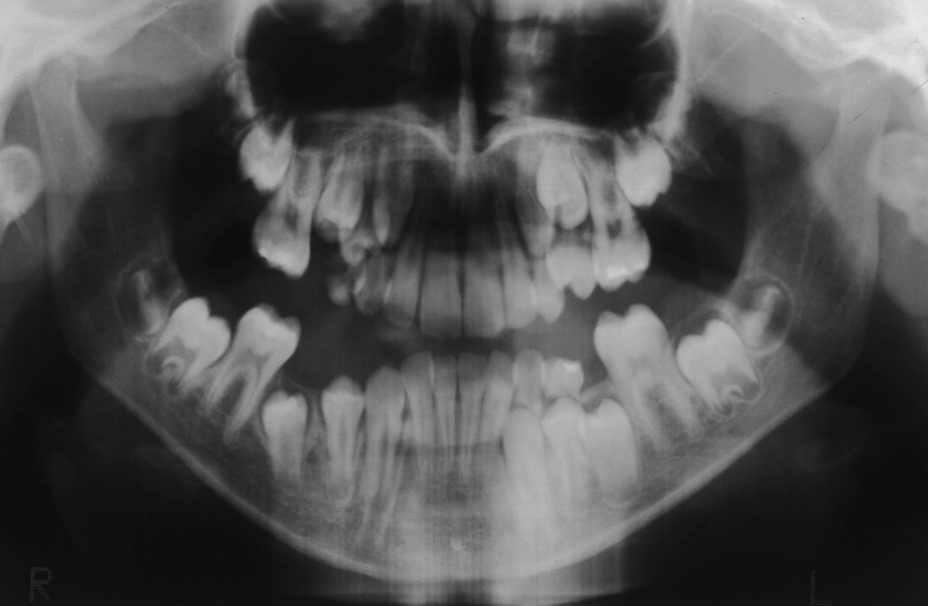
**Panoramic radiographic view demonstrating chronologically delayed eruption and radicular abnormalities of the second molars**.

**Figure 5:**
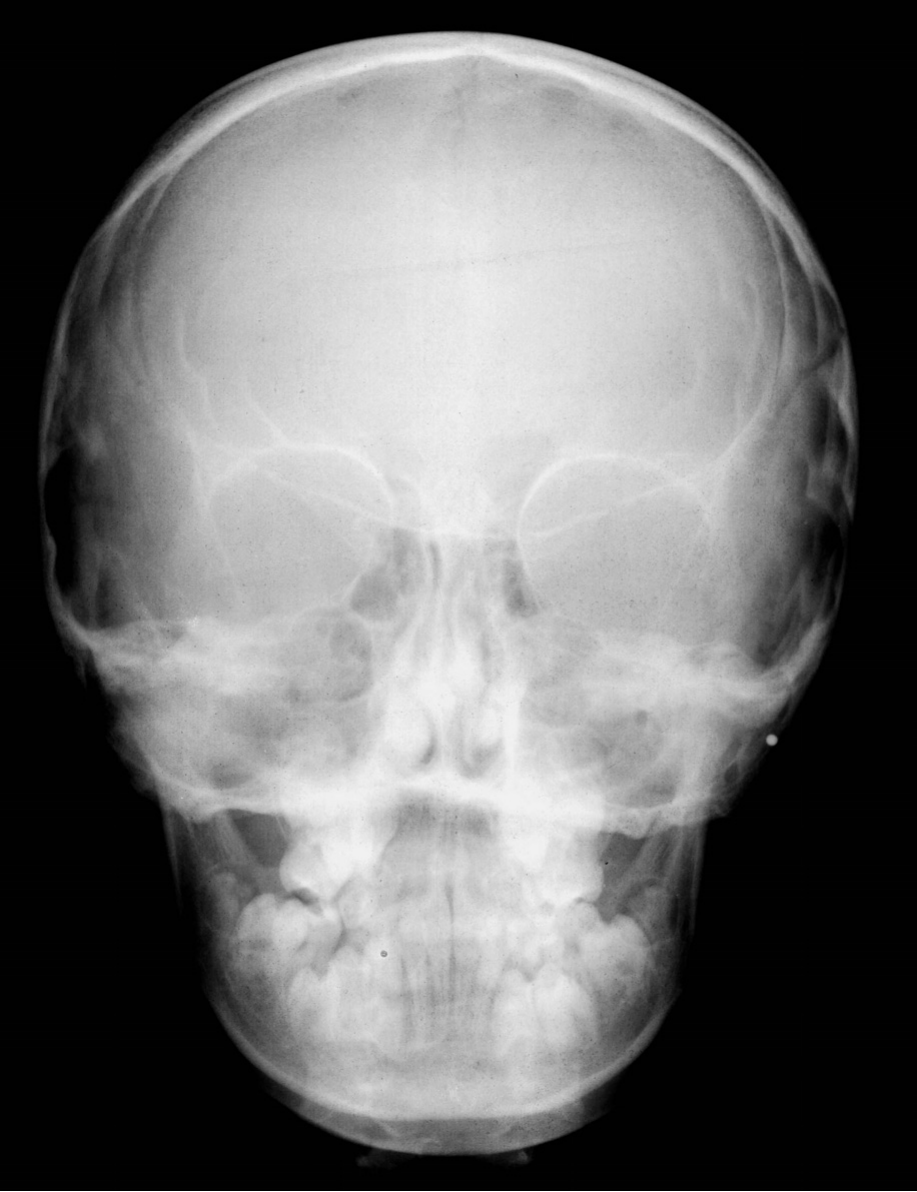
**Anteroposterior X-ray of the skull showing hypoplastic frontal sinuses and mild nasal septum deviation**.

**Figure 6:**
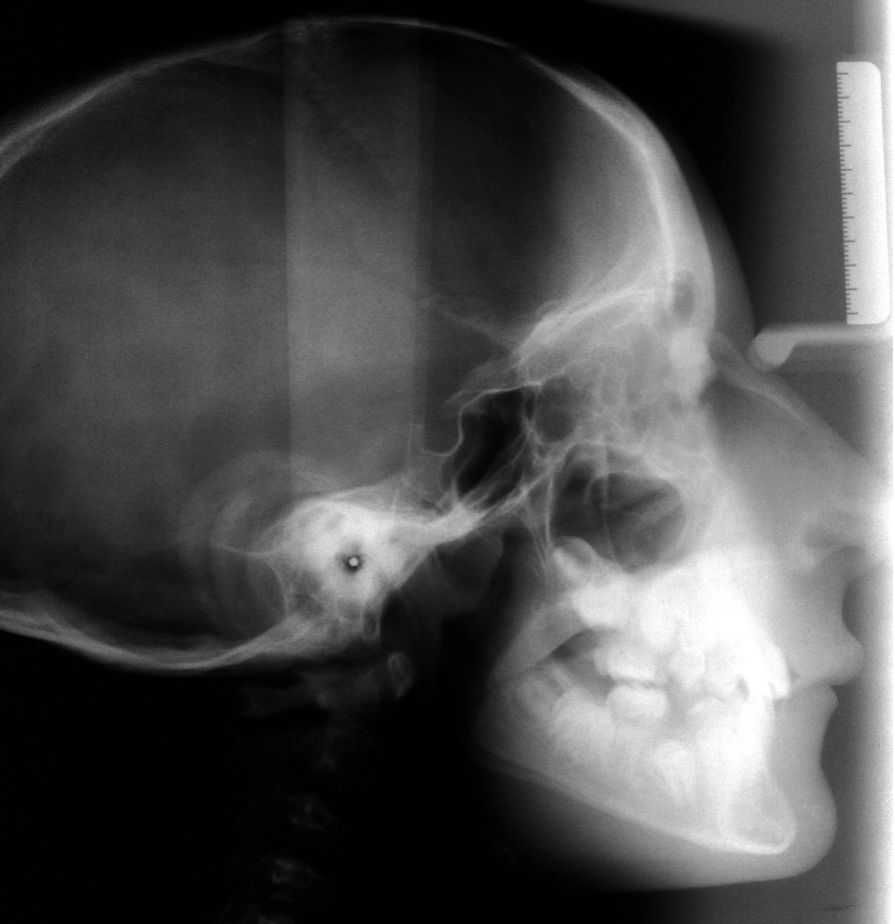
**Lateral radiography views of the skull, with skeletal class II, ethmoid cell hypoplasia, frontal bone thickness, normal maxillary sinus and mild frontal hyperostosis**.

Based on the panoramic radiograph, our patient presented with chronologically delayed eruption and radicular abnormalities of the second molars (Figure 4). Anteroposterior radiological sinonasal findings showed hypoplastic frontal sinuses and mild nasal septum deviation (Figure 5). In addition lateral radiography views of our patient's skull showed skeletal class II, ethmoid cell hypoplasia, frontal bone thickness, a normal maxillary sinus and mild hyperostosis frontalis (Figure 6).

## Discussion

The overall incidence of DS has not been established, but it is very rare. Approximately 150 cases have been reported in the literature, with various other associated anomalies [1,2]. Most of the cases have been reported in the USA, Europe, Middle East and Russia, as well as Japan [8]. It appears to affect both sexes and all ethnicities equally.

The most common physical characteristics associated with DS are growth retardation, a characteristic facial appearance and microcephaly [9,10]. Growth retardation is usually due to growth hormone (GH) deficiency, and could be due to gene mutations or disruption of brain structures during development [10,11]. GH deficiency also has a correlation with low levels of IgG, which are also found in patients with DS [11]. An MRI study of a patient with DS with GH deficiency revealed congenital midline abnormalities including corpus callosum dysgenesis, and hypoplastic anterior pituitary gland and stalk, with an ectopic neurohypophysis [12].

The diagnostic phenotypic features of DS in our patient included small low-set ears, saddle nose, triangular face, mental retardation, abnormal high-pitched voice, hypertelorism, cataract, cardiological features and scoliosis. In a review on 141 individuals with DS, facial anomalies were suggested to be the most diagnostic of the physical signs [1]. Of the 141 patients, 15 had a normal appearance. Microcephaly was present in 112 patients, blepharophimosis in 60 and ptosis in 53. The authors considered a prominent round nose tip, noted in 17 of their 34 cases, to be especially characteristic of DS at a young age.

To the best of our knowledge, our case report is only the second study to detail the specific oral features of a single patient [13]. A wide array of characteristics can be present. Multiple dental carious lesions are found in the majority of cases [1]. Other dental features include retarded eruption, microdontia, malocclusion, diastema and fusion of dental elements, and anodontia of the central incisors is generally present [1]. Oral features include a small oral cavity, thin upper lip border, prominent philtrum, narrow and deep palate, palatine cleft, submucosal palatine cleft, split uvula, micrognathia, prognathism and retrognathism [1,2] In 1990, velopharyngeal insufficiency was described for the first time [14]. Our patient presented with the typical oral alterations of cleft palate, incipient cavities, retarded eruption and malocclusion.

Other physical problems caused by DS, such as blepharoptosis or cardiovascular defects, can be corrected through surgery [10]. A number of behavioral characteristics have been reported by parents of children with DS, and described in the medical literature [2,7,15-17]. These include extreme hyperactivity and language difficulties [17,18].

DS has autosomal recessive inheritance [15]. In 2 of 15 familial cases, the parents were consanguineous [19,20]. Several cases of DS have been found to occur in monozygotic twins, siblings and cousins [6]. Affected siblings have been described in nine families, with both sexes affected. One set of concordantly affected monozygotic twins has been reported. In a set of dizygotic twins, only one twin was affected [1]. A few authors have suggested that DS may represent another disorder caused by an alteration in sterol synthesis, transport or metabolism [21]. Recently, a case of DS with persistently low cholesterol levels has been described [22]. This finding correlates with findings in Smith-Lemli-Opitz syndrome (SLOS), one of the conditions considered in the differential diagnosis of DS. Patients with SLOS and DS have common clinical features, and both conditions have therefore been hypothesized to be linked to a defect in the cholesterol biosynthetic pathway [21,22]. SLOS is caused by mutations in the *DHCR7 *gene, which makes an enzyme called 7-dehydrocholesterol reductase [23]. Mutations in the *DHCR7 *gene reduce or eliminate the activity of this enzyme, preventing cells from producing sufficient cholesterol [23,24]. A lack of this enzyme also allows potentially toxic byproducts of cholesterol production to build up in the blood and other tissues [24]. The combination of low cholesterol levels and an accumulation of other substances is likely to disrupt the growth and development of many body systems [24]. However, it is not known how this disturbance in cholesterol production leads to the specific features of SLOS [23,24].

Although there is considerable evidence pointing to the genetic basis of this disorder, the symptoms that are expressed are very similar to fetal alcohol syndrome (FAS), and further studies need to be performed to determine whether this environmental agent has an effect on the expression of the genotype [16]. Clinically, children diagnosed with FAS vary greatly in symptom presentation, probably due to the amount of alcohol and timing of exposure, as well as maternal and genetic influences; however, no genetic markers have yet been found, except in mouse models [25-28]. All these factors play a role in determining the mechanisms through which alcohol damages the developing brain, the details of which are still largely unknown [26,28,29].

One of the symptoms of DS is the breakdown of chromosomes [24], and it therefore needs to be differentiated from Bloom syndrome (BS) [1,2]. BS is the prototype of the class of human diseases referred to as 'chromosome breakage syndromes' [30]. The cytogenetic features of BS cells in mitosis are increased numbers of chromatid gaps, breaks and rearrangements, and increased numbers of quadriradial configurations [31]. A greatly increased frequency of sister chromatid exchanges in cells exposed to bromodeoxyuridine is diagnostic; BS is the only disorder in which such evidence of hyper-recombination is known to occur [32]. Mutations in the *BLM *gene, which is a member of the DNA helicase family, are associated with BS [33-38]. DNA helicases are enzymes that unwind the two strands of a duplex DNA molecule [33,34]. A second mutation segregating among the Ashkenazi Jewish population, insT2407, has been identified (Bloom's Syndrome Registry, unpublished data). The greatly elevated rate of mutation in BS results in a high risk of cancer in affected individuals [39]. The cancer predisposition is characterized by (i) a wide range of cancer types, including leukemias, lymphomas, and carcinomas; (ii) an early age of onset relative to the same cancer in the general population; and (iii) multiplicity [40]. The average age of cancer diagnosis in patients with BS is approximately 25 years, but cancer may develop at any age. A previous report has described co-occurrence of embryonal rhabdomyosarcoma and multiple spontaneous chromosome breaks; in that case, the tumor was resected, but recurred, resulting in the child's death at three months of age [37].

## Conclusion

Because the genetic cause of BS is not known, there is no specific medical test that can definitively assign the diagnosis. The diagnosis is usually based on the characteristic facial appearance of the affected individual, and on other factors such as growth data and medical history. The diagnosis is easily missed if the physician is not familiar with genetic pediatric conditions. Early diagnosis is essential, as the prognosis for patients with DS is good provided that management of their medical conditions is initiated early and maintained throughout life. Patients with DS can be expected to survive to adulthood and lead a fairly normal lifestyle, although most have some level of mental retardation. DS involves various systems, including the stomatognathic system, emphasizing one of the reasons why it is important for health professionals to recognize the characteristics and consequently refer such patients for the necessary multidisciplinary treatments. Microarray studies may be useful in the identification of a genetic marker for DS syndrome or for the discovery of novel pathways that may be involved in its origin.

## Consent

Written informed consent was obtained from the patient's parents for publication of this case report and any accompanying images. A copy of the written consent is available for review by the Editor-in-Chief of this journal.

## Competing interests

The authors declare that they have no competing interests.

## Authors' contributions

SC and DT were responsible for the clinical follow-up of our patient. AB and AD edited and coordinated the manuscript. All authors read and approved the final manuscript.
